# Supplementary material for: Magnetic Isolation of the Linear Trinuclear Anion in [Cu(Him)6] {Cu(Him)4[Cu(μ-EDTA)(Him)]2}·6H2O (1) as the Novel Imidazolium(+) Salt (H2im)2[Cu(Him)4{(µ-EDTA)Cu(Him)}2]·2H2O (2)—A Comparative Look to Their Crystal Structures, Thermal, Spectral and Magnetic Properties and DFT Calculations
Source: Int J Mol Sci. 2024 Dec 6;25(23):13130. doi: 10.3390/ijms252313130 (PMC11641978; doi:10.3390/ijms252313130)
Supplement: Supplementary file 1 [file ijms-25-13130-s001.zip › ijms-3341277-supplementary.pdf]

Supplementary Materials

for

Magnetic isolation of the linear trinuclear anion in $[Cu(Him)_6]\{\mu\text{-}Cu(Him)_4[Cu(EDTA)(Him)]_2\}\cdot 6H_2O$ (1) as the novel imidazolium(+) salt $(H_2im)_2[Cu(Him)_4\{(\mu\text{-}EDTA)Cu(Him)\}_2]\cdot 2H_2O$ (2).
A comparative look to their crystal structures, thermal, spectral and magnetic properties and DFT calculations.

Jeannette Carolina Belmont-Sánchez,[1] Duane Choquesillo-Lazarte,[2] Antonio Frontera[3], Luis Lezama,[4] Alfonso Castiñeiras,[5] Juan Niclós-Gutiérrez,[2,*]

1 Department of Inorganic Chemistry, Faculty of Pharmacy, University of Granada, 18071 Granada, Spain; E-mails: jniclos@ugr.es, carol.bs.quimic@hotmail.com
2 Laboratorio de Estudios Cristalográficos, IACT, CSIC-Universidad de Granada, Av. de las Palmeras 4, E-18100 Armilla, Granada, Spain; E-: mail: duane.choquesillo@csic.es
3 Department de Química, Universitat de les Illes Balears, Crta. de Valldemossa km 7.5, 07122 Palma de Mallorca, Spain; E-mail: toni.frontera@uib.es
4 Department of Inorganic Chemistry, Faculty of Science and Technology, University of Basque Country, E-48080 Bilbao, Spain; E-mail: luis.lezama@ehu.es
5 Department of Inorganic Chemistry, Faculty of Pharmacy, University of Santiago de Compostela, 15782 Santiago de Compostela, Spain; Email: alfonso.castineiras@usc.es
* Correspondence: jniclos@ugr.es

S.1	Additional Structural Information of Compound **1**
S.2	Additional Structural Information of Compound **2**
S.3	Plots for the Conformational Analysis of the Pentadentate μ-EDTA in **1** and **2**
S.4	TGA studies of Compounds 1 and 2
S.5	FT-IR spectra of solid samples (as KBr disks) for Compounds **1** and **2**

S.1 Additional Structural Information of Compound 1

These data are obtained from the cif file GEMPOE deposited in the CSD database (see Ref. 6 in the Manuscript).

Table S1. Selected bond lengths [Å] and some relevant angles [°] for [Cu(Him)$_6$]{μ-Cu(Him)$_4$[Cu(EDTA)(Him)]$_2$}·6H_2O (**1**)

Cu(1)-N(3)#2	2.0206(15)
Cu(1)-N(3)	2.0206(15)
Cu(1)-N(5)#2	2.0559(14)
Cu(1)-N(5)	2.0559(14)
Cu(1)-N(1)	2.4684(15)
Cu(1)-N(1)#2	2.4684(15)
Cu(2)-N(13)	1.9849(14)
Cu(2)-N(13)#1	1.9849(14)
Cu(2)-N(11)#1	2.0228(14)
Cu(2)-N(11)	2.0229(14)
**Cu(2)-Cu(3)**	**6.084(3)**
**Cu(2)-Cu(1)**	**15.155(6)**
Cu(3)-O(3)	1.9654(13)
Cu(3)-O(5)	1.9684(13)
Cu(3)-N(7)	1.9681(15)
Cu(3)-N(10)	2.0715(15)
Cu(3)-O(1)	2.3810(12)
Cu(3)-N(9)	2.3982(14)
Cu(3)-Cu(1)	9.724(4)
N(3)#2-Cu(1)-N(3)	180.0
N(3)#2-Cu(1)-N(5)#2	89.99(6)
N(3)-Cu(1)-N(5)#2	90.01(6)
N(3)#2-Cu(1)-N(5)	90.01(6)
N(3)-Cu(1)-N(5)	89.99(6)
N(5)#2-Cu(1)-N(5)	180.0
N(3)#2-Cu(1)-N(1)	90.46(5)
N(3)-Cu(1)-N(1)	89.54(6)
N(5)#2-Cu(1)-N(1)	93.76(5)
N(5)-Cu(1)-N(1)	86.24(5)
N(3)#2-Cu(1)-N(1)#2	89.54(6)
N(3)-Cu(1)-N(1)#2	90.46(6)
N(5)#2-Cu(1)-N(1)#2	86.24(5)
N(5)-Cu(1)-N(1)#2	93.75(5)

N(1)-Cu(1)-N(1)#2	180.0
N(13)-Cu(2)-N(13)#1	180.00(6)
N(13)-Cu(2)-N(11)#1	91.61(6)
N(13)#1-Cu(2)-N(11)#1	88.40(6)
N(13)-Cu(2)-N(11)	88.40(6)
N(13)#1-Cu(2)-N(11)	91.60(6)
N(11)#1-Cu(2)-N(11)	180.0
O(3)-Cu(3)-O(5)	170.99(4)
O(3)-Cu(3)-N(7)	89.12(6)
O(5)-Cu(3)-N(7)	95.46(6)
O(3)-Cu(3)-N(10)	84.52(6)
O(5)-Cu(3)-N(10)	91.71(6)
N(7)-Cu(3)-N(10)	171.03(5)
O(3)-Cu(3)-O(1)	96.34(5)
O(5)-Cu(3)-O(1)	90.73(5)
N(7)-Cu(3)-O(1)	97.87(5)
N(10)-Cu(3)-O(1)	76.62(5)
O(3)-Cu(3)-N(9)	92.67(5)
O(5)-Cu(3)-N(9)	78.63(5)
N(7)-Cu(3)-N(9)	105.67(5)
N(10)-Cu(3)-N(9)	81.00(5)
O(1)-Cu(3)-N(9)	154.90(4)

Symmetry transformations used to generate equivalent atoms:
#1 -x+2,-y+2,-z+1, #2 -x+1,-y,-z+2

Table S2. Conventional H-bonds for [Cu(Him)$_6$]{μ-Cu(Him)$_4$ [Cu(EDTA(Him]$_2$·6H$_2$O (1) [Å and °].

D–H···A	δ(D···A)	∠(DHA)
N(8)-H(40)···O(11)#3	2.7517(18)	175.9
N(12)-H(41)···O(9)	2.7637(19)	166.0
N(14)-H(42)···O(10)#4	2.7477(18)	159.0
C(10)-H(10)···O(2)#5	3.274(2)	148.9
C(24)-H(16)···O(6)	2.988(2)	121.1
C(26)-H(18)···O(5)#1	3.366(2)	163.2
C(27)-H(27)···O(8)	3.0600(19)	111.9
N(2)-H(37)···O(1)#3	3.1221(19)	168.1
N(4)-H(38)···O(7)	2.733(2)	172.2
N(6)-H(39)···O(2)#6	2.7261(18)	177.8
C(4)-H(4)···N(1)	3.166(2)	119.2
O(9)-H(31)···O(10)#7	2.7934(19)	172.1
O(9)-H(32)···O(8)#8	2.6864(18)	142.8
O(10)-H(33)···O(4)	2.6503(17)	153.2
O(11)-H(35)···O(1)#6	2.8855(17)	165.1
O(11)-H(35)···O(2)#6	3.283(2)	132.1
O(11)-H(36)···O(3)	2.846(2)	170.2

Symmetry transformations used to generate equivalent atoms:
#1 -x+2,-y+2,-z+1 #2 -x+1,-y,-z+2 #3 -x+1,-y+1,-z+2
#4 -x+2,-y+1,-z+1 #5 x-1,y,z #6 -x+2,-y+1,-z+2
#7 x-1,y+1,z #8 -x+1,-y+2,-z+1

S.2 Additional Structural Information of Compound 2

Table S3. Crystal data and structure refinement for $(H_2im)_2[Cu(Him)_4\{(\mu\text{-}EDTA)Cu(Him)\}_2]\cdot 2H_2O$ (**2**).

Empirical formula	C_{44} H_{62} Cu_3 N_{20} O_{18}
Formula weight	1349.75
Temperature	298(2) K
Wavelength	1.54178 Å
Crystal system, space group	Triclinic, $P\bar{1}$
Unit cell dimensions	
a = 8.3552(2) Å	α = 71.4260(10) °
b = 12.2097(3) Å	β = 75.6010(10) °
c = 16.0405(3) Å	γ = 71.3990(10) °
Volume	1450.27(6) $Å^3$
Z, Calculated density	1, 1.545 Mg/m^3
Absorption coefficient	2.023 mm^{-1}
F(000)	697
Crystal size	0.12 × 0.10 × 0.10 mm
Theta range for data collection	2.946 to 66.574 °
Limiting indices, *h, k, l*	-9/9, -13/14, -19/19
Reflections collected / unique	21499 / 4980 [R_{int} = 0.0310]
Completeness to theta	67.679 (95.1 %)
Absorption correction	Semi-empirical from equivalents
Max. and min. transmission	1.000 and 0.890
Refinement method	Full-matrix least-squares on F^2
Data / restraints / parameters	4980 / 0 / 395
Goodness-of-fit on F^2	1.097
Final *R* indices [I>2σ(I)]	R_1 = 0.0425, wR_2 = 0.1170
R indices (all data)	R_1 = 0.0460, wR_2 = 0.1202
Largest diff. peak and hole	0.376 and −0.588 e.$Å^{-3}$

Table S4. Selected bond lengths [Å] and trans-angles [°] in the crystal of $(H_2im)_2[Cu(Him)_4\{(\mu\text{-EDTA})Cu(Him)\}_2]\cdot 2H_2O$ (**2**).

Cu(1)-O(4)	1.9497(19)
Cu(1)-O(15)	1.9737(18)
Cu(1)-N(20)	1.988(2)
Cu(1)-N(1)	2.102(2)
Cu(1)-N(12)	2.326(2)
Cu(1)-O(8)	2.456(2)
**Cu(1)-Cu(2)**	**6.8025(4)**
Cu(2)-N(30)	1.996(2)
Cu(2)-N(30)#1	1.996(2)
Cu(2)-N(25)	2.033(2)
Cu(2)-N(25)#1	2.033(2)
Cu(2)-O(19)#1	2.720(3)
Cu(2)-O(19)	2.720(3)
O(4)-Cu(1)-O(15)	175.75(8)
O(4)-Cu(1)-N(20)	88.63(9)
O(15)-Cu(1)-N(20)	95.16(9)
O(4)-Cu(1)-N(1)	84.62(8)
O(15)-Cu(1)-N(1)	91.95(8)
N(20)-Cu(1)-N(1)	168.44(9)
O(4)-Cu(1)-N(12)	96.40(8)
O(15)-Cu(1)-N(12)	80.62(7)
N(20)-Cu(1)-N(12)	107.86(9)
N(1)-Cu(1)-N(12)	82.27(8)
O(4)-Cu(1)-O(8)	95.43(9)
O(15)-Cu(1)-O(8)	85.91(8)
N(20)-Cu(1)-O(8)	98.87(9)
N(1)-Cu(1)-O(8)	72.52(8)
N(12)-Cu(1)-O(8)	150.93(7)

N(30)-Cu(2)-N(30)#1	180.0
N(30)-Cu(2)-N(25)	90.20(9)
N(30)#1-Cu(2)-N(25)	89.80(10)
N(30)-Cu(2)-N(25)#1	89.80(10)
N(30)#1-Cu(2)-N(25)#1	90.20(9)
N(25)-Cu(2)-N(25)#1	180.00(8)
N(30)-Cu(2)-O(19)#1	91.21(9)
N(30)#1-Cu(2)-O(19)#1	88.79(9)
N(25)-Cu(2)-O(19)#1	80.79(9)
N(25)#1-Cu(2)-O(19)#1	99.21(9)
N(30)-Cu(2)-O(19)	88.79(9)
N(30)#1-Cu(2)-O(19)	91.21(9)
N(25)-Cu(2)-O(19)	99.21(9)
N(25)#1-Cu(2)-O(19)	80.79(9)
O(19)#1-Cu(2)-O(19)	180.0

Symmetry transformations used to generate equivalent atoms: #1 -x,-y+2,-z

Intermetallic distances < 10 Å n the crystal of compound 2

Coordination Sphere Around Cu(1) (Å)

**1**	**6.8025**	**Cu(2)**
2	7.9056	Cu(2)e [1+x,-1+y,z]
3	7.9836	Cu(1)g [1-x,1-y,-z]
4	8.3552	Cu(1)b [-1+x,y,z]
5	8.3552	Cu(1)c [1+x,y,z]
6	8.4425	Cu(1)h [-x,1-y,1-z]
7	8.6775	Cu(2)d [x,-1+y,z]
8	9.6990	Cu(1)f [-x,1-y,-z]
9	9.9274	Cu(2)c [1+x,y,z]

Coordination Sphere Around Cu(2) (Å)

**1**	**6.8025**	**Cu(1)a [-x,2-y,-z]**
**2**	**6.8025**	**Cu(1)**
3	7.9056	Cu(1)g [1-x,1-y,-z]
4	7.9056	Cu(1)c [-1+x,1+y,z]
5	8.3552	Cu(2)b [-1+x,y,z]
6	8.3552	Cu(2)e [1+x,y,z]
7	8.6775	Cu(1)f [-x,1-y,-z]
8	8.6775	Cu(1)d [x,1+y,z]
9	9.9274	Cu(1)b [-1+x,y,z]
10	9.9274	Cu(1)h [1-x,2-y,-z]

Table S5. Hydrogen bonds for $(H_2im)_2[Cu(Him)_4\{(\mu\text{-EDTA})Cu(Him)\}_2]\cdot 2H_2O$ (**2**) [Å and °].

D–H···A	δ(D···A)	∠(DHA)
N(22)-H(22)...O(5)#2	2.804(3)	177.9
N(27)-H(27)...O(19)#3	2.893(4)	169.6
N(32)-H(32)...O(8)#4	2.701(3)	151.2
N(35)-H(35)...O(15)	3.178(3)	120.5
N(35)-H(35)...O(16)	2.672(3)	175.6
N(37)-H(37)...O(9)#5	2.642(4)	158.6
O(1A)-H(1)...O(20)#6	2.756(5)	174.2
O(1A)-H(2)...O(9)	2.833(5)	162.1
O(1B)-H(3)...O(9)	2.753(11)	141.9
O(1B)-H(4)...O(20)#6	2.737(5)	147.6
C(2)-H(2A)...O(16)#3	3.523(4)	164.2
C(2)-H(2B)...O(1A)#7	3.585(9)	170.1
C(11)-H(11A)...O(20)	3.065(4)	114.8
C(13)-H(13A)...O(5)#8	3.358(4)	172.3
C(28)-H(28)...O(1A)#7	3.264(6)	144.8
C(28)-H(28)...O(1B)#7	3.283(12)	168.9
C(31)-H(31)...O(20)	3.225(4)	159.2
C(34)-H(34)...O(19)#1	3.261(4)	123.8
C(39)-H(39)...O(1A)#8	3.251(6)	151.8
C(39)-H(39)...O(1B)#8	3.440(18)	147.2

Symmetry transformations used to generate equivalent atoms: #1 -x,-y+2,-z #2 -x+1,-y+1,-z #3 x+1,y,z #4 x-1,y+1,z #5 -x+1,-y,-z+1 #6 -x,-y+1,-z+1 #7 -x+1,-y+1,-z+1 #8 x-1,y,z

Table S6. Intermolecular π···π interaction parameters (Å, º)

**Compound**	**π···π**	**Symmetry**	**Cg(I)···Cg(J)**	**α**	**β**	**γ**
**2**	Cg(1)···Cg(1)^i	i = *-x, -y, 1-z*	3.457	0.00	3.81	3.81

Cg(I)···Cg(J): Distance between ring centroids; Cg(1) is the centroid of the N(35)/C(36)/N(37)/C(38/C(39) ring; α: Dihedral angle between planes I and J. for details, see Janiak, C. (2000). J. Chem. Soc. Dalton Trans. pp. 3885–3898.

S.3 Plots for the Conformational Analysis of the Pentadentate μ-EDTA in 1 and 2.

Caution: The purpose of these figures is to illustrate that the chelating and bridging ligand μ-EDTA adopts its most stable conformation in both compounds, 1 and 2. Thus, with respect to the P[N-Cu-N] plane of the ethylene unit, its C atoms are outside this plane and each one on opposite sides.
Since in both, 1 and 2, their linear trinuclear anions are centro symmetrics, their μ-EDTA chelators of the terminal Cu centers are equivalent. So the different color used for the P[N-Cu-N] planes only has an aesthetic reason.

Compound 1 (water molecules and H-atoms omitted)

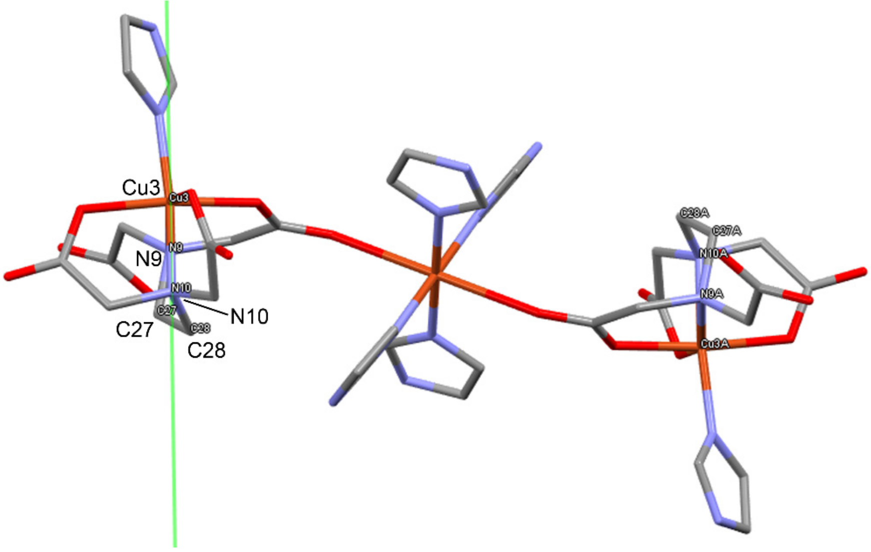

Compound 2 (water molecules and H-atoms omitted)

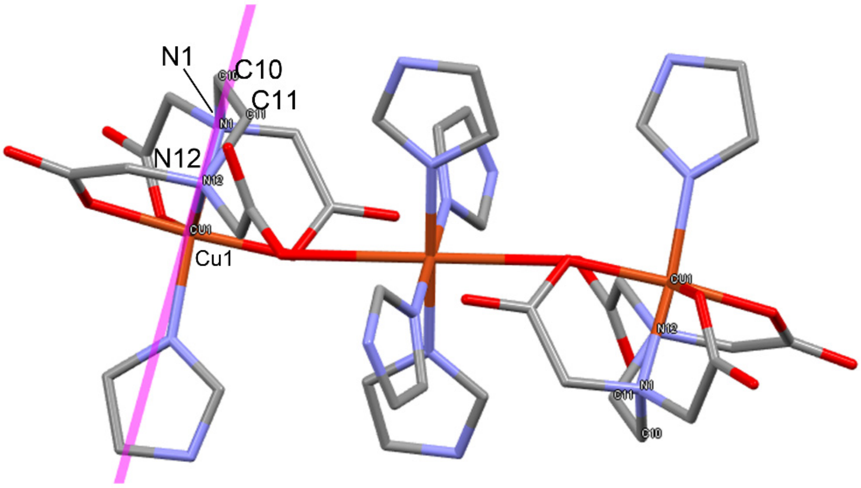

COMPLEMENTARY LITERATURE

1. Smith, G.S.; Hoard, J. L. The Structure of Dihydrogen Ethylenediaminetetraacetato-aquonickel(II).
J. Am. Chem. Soc. **1959**, *81*, 556-561. https://pubs.acs.org/**doi**/10.1021/ja01512a012

2. Kocanova, I.; Kuchar, J.; Dankovicova, V.; Cernak, J. Redetermination of aqua-(dihydrogen ethyl-enediamine-tetra-acetato-κO,O',N,N',O'')nickel(II). Acta Crystallogr., Sect.E:, **2010**, *66*, m196-m1967. https://doi:10.1107/S1600536810002011
3. F. P. Dwyer and D. P. Mellor (editors), Chelating Agents and Metal Chelates. Academic Press Inc., 1964. F.L. Garvan (Chapter 7): Metal Chelates of Ethylenediaminotetraacetic acid and Related Substances.
4. Von Zelewsky, A. Stereochemistry of Coordination Compounds**,** John Wiley & Son Ltd. New York, 1996. Chapter 5. Topographical Stereochemistry of Mononuclear Coordination, Units. pp. 116-128.
5. Hawkins, C.J. Absolute Configuration of Metal Complexes, John Wiley & Son Ltd. New York, 1971. Chapters 2 (Notation of Absolute Configuration) and 3 (Conformational Analysis).

S.4 TGA studies of 1 and 2

S.4.1 Compound 1 (GEMPOE in CSD)

TGA plot

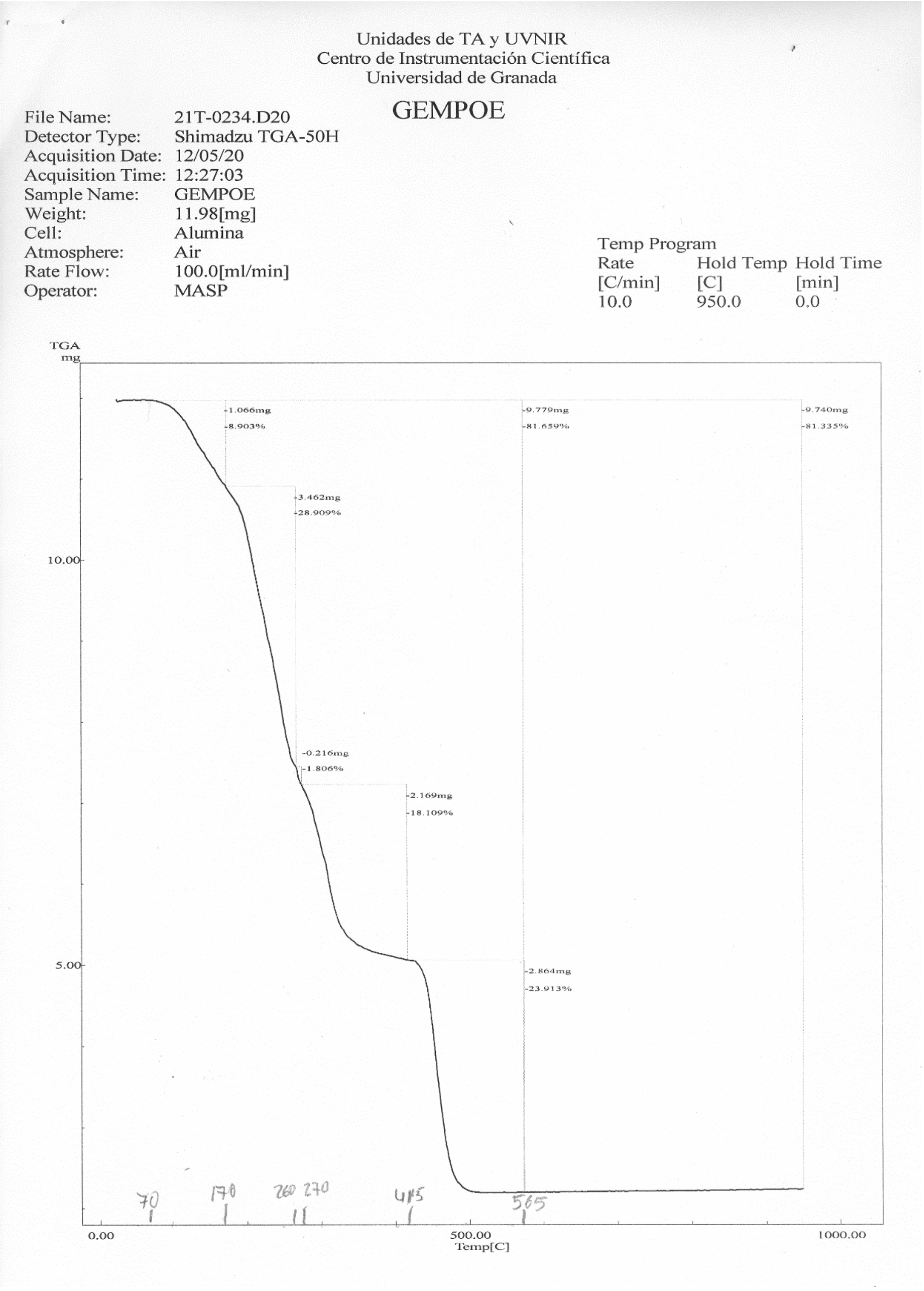

Three selected FT-IR images to identify the evolved gases at steps 4 (IV) and 5 (V), and to the end of the experiment. The non-identified gases are, once more and clearly, detected at 850-950 cm^{-1}. In these images, commas are used as decimal separators instead of dots.

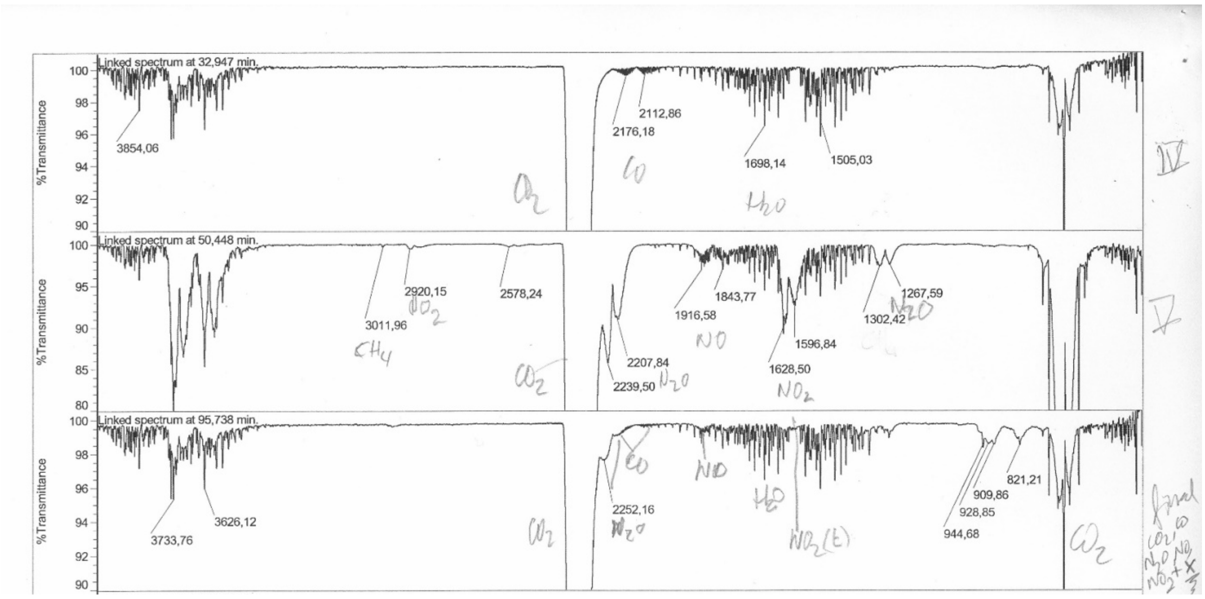

Plot of the FT-IR spectra, from the IR library, used for the identification of evolved gases during the TGA of compound **1** (GEMPOE in CSD).

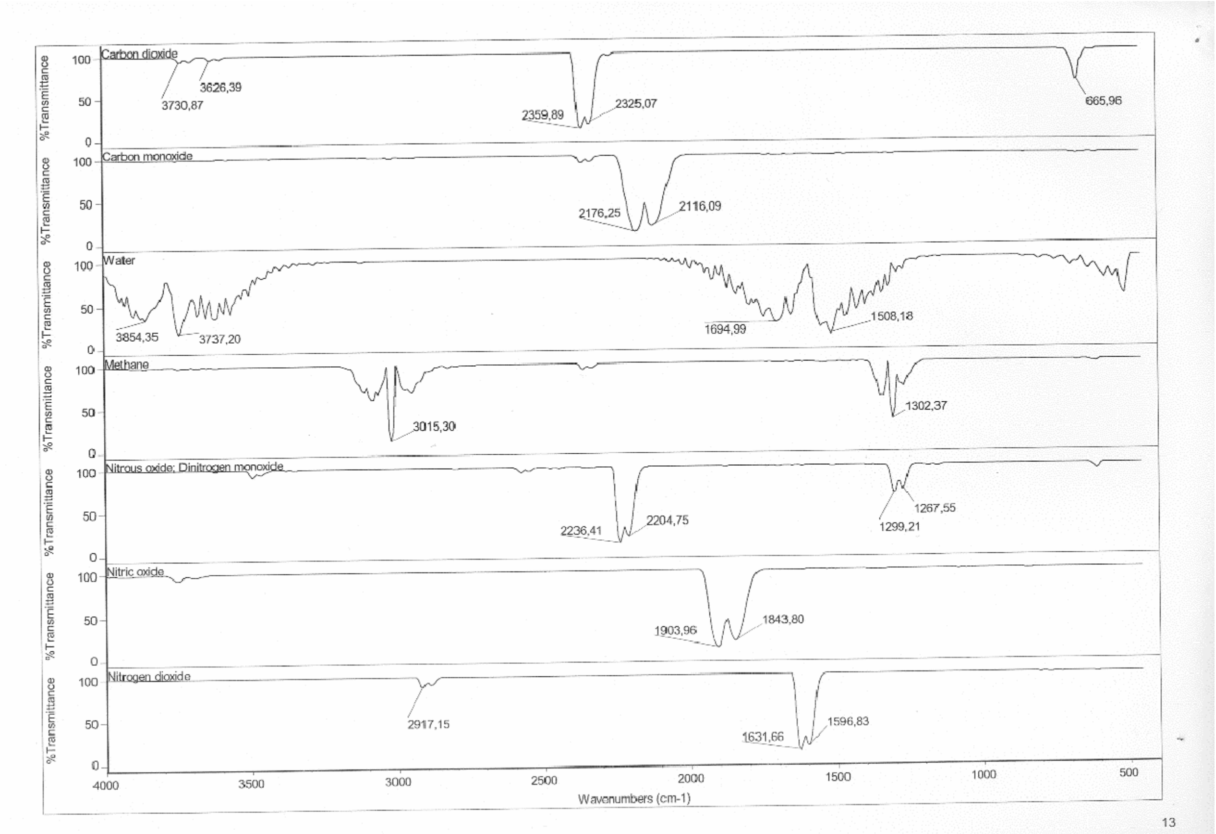

S.4.2 Compound 2 (internal code C-966)

TGA plot

Unidades de TA y UVNIR
Centro de Instrumentación Científica
Universidad de Granada

C-966

File Name:	21T-0233.D20
Detector Type:	Shimadzu TGA-50H
Acquisition Date:	12/05/20
Acquisition Time:	08:33:23
Sample Name:	C-966
Weight:	13.26[mg]
Cell:	Alumina
Atmosphere:	Air
Rate Flow:	100.0[ml/min]
Operator:	MASP

Temp Program

Rate	Hold Temp	Hold Time
[C/min]	[C]	[min]
10.0	950.0	0.0

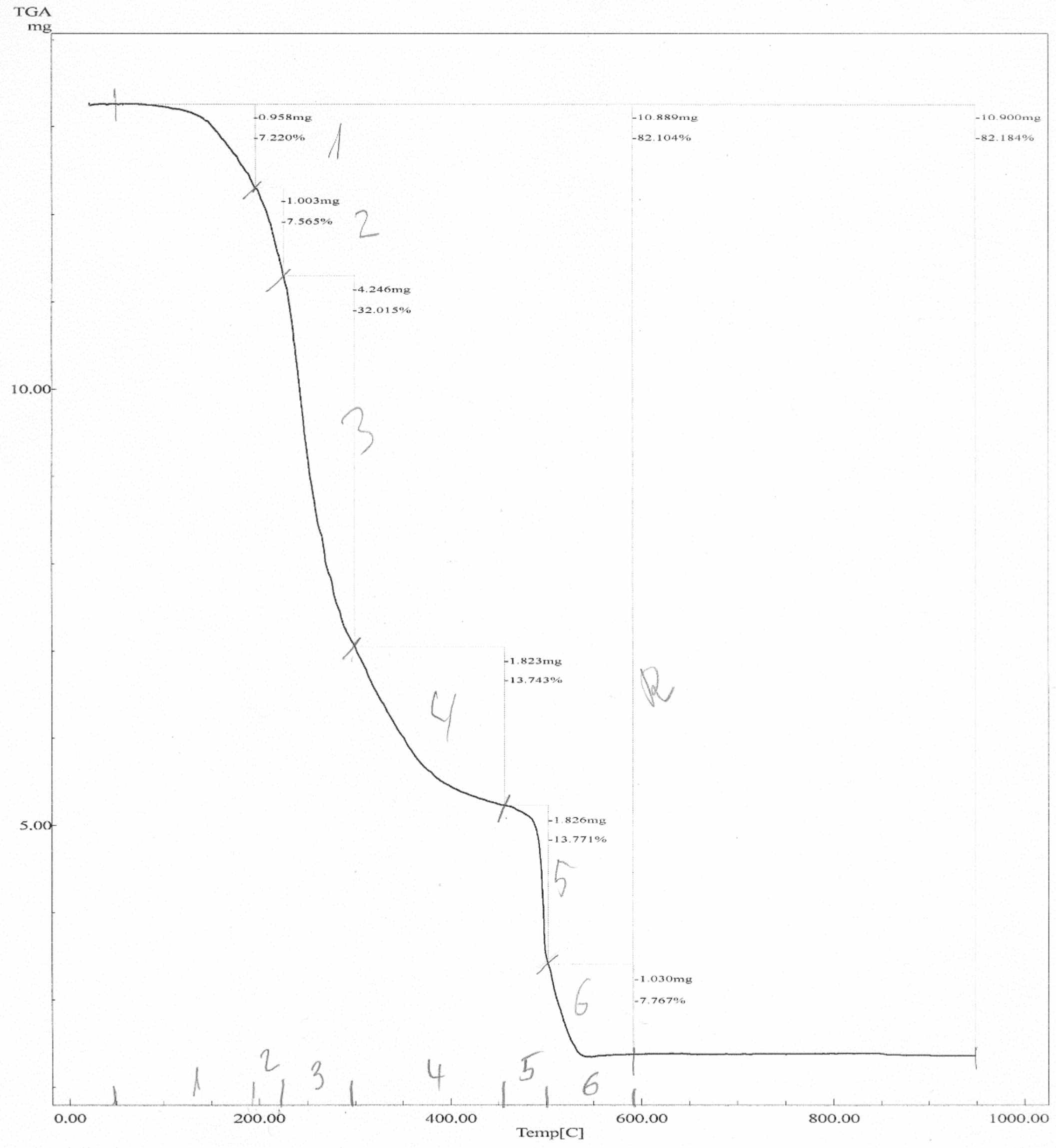

4

Thermogravimetric analyses (TGA) with identification of evolved gasses by FT-IR library for **compound 2**. MW: (1) = 1349.73, H_2O = 18.015, CuO = 79.545.

Step or R	Temp. (°C)	Time (min)	Weight (%) Exp.	Cal.	Evolved gases or residue (R)
1	50-190	2-17	7.220	>>2.669	2 H_2O (N), CO_2
2	190-220	17-20	7.565	-	CO_2, H_2O
3	220-295	20-25	32.015		CO_2, H_2O, CO(t)
4	295-455	25-44	13.743		CO_2, H_2O, CO, N_2O
5	455-500	44-47	13.771	-	CO_2, H_2O, CO, N_2O, NO, CH_4
6	500-580	47-60	7.767		CO_2, H_2O, CO, N_2O, NO, NO_2,CH_4, X
R1	580	60	17.896	17.680	CuO (with some impurities)
R2	950	93	17.852	17.680	CuO

R = residue(s). N = undetermined value. t = trace amounts. X = unidentified gas(es).

In these images, commas are used as decimal separators instead of dots.

Plots with sequential FT-IR spectra of the evolved gases along with all the TGA experiment of the novel Compound 2.

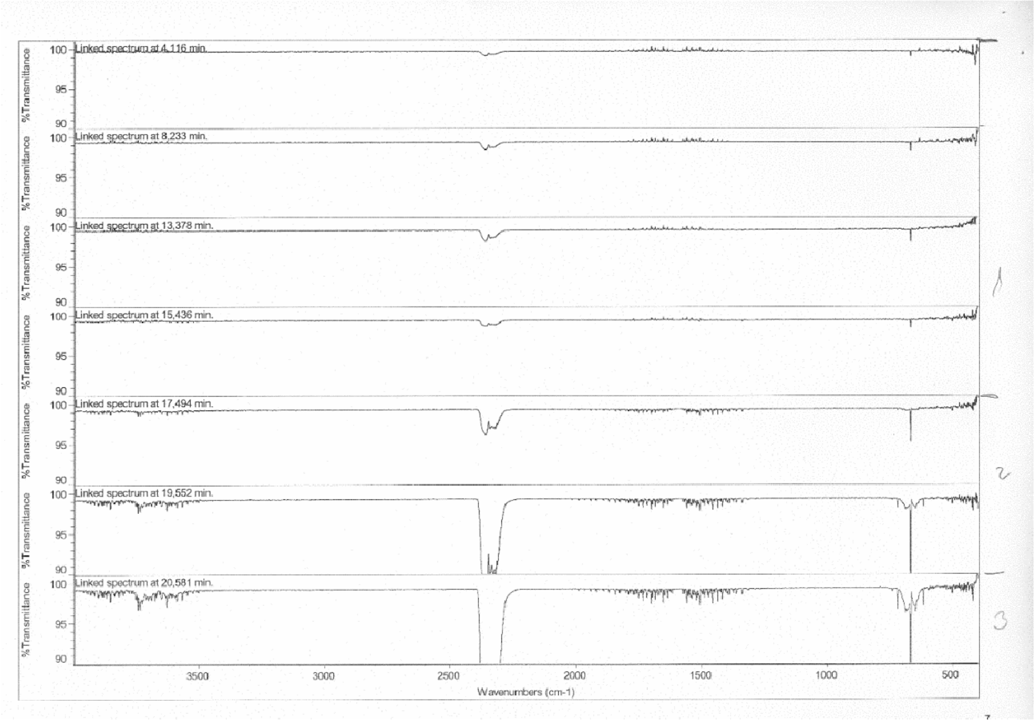

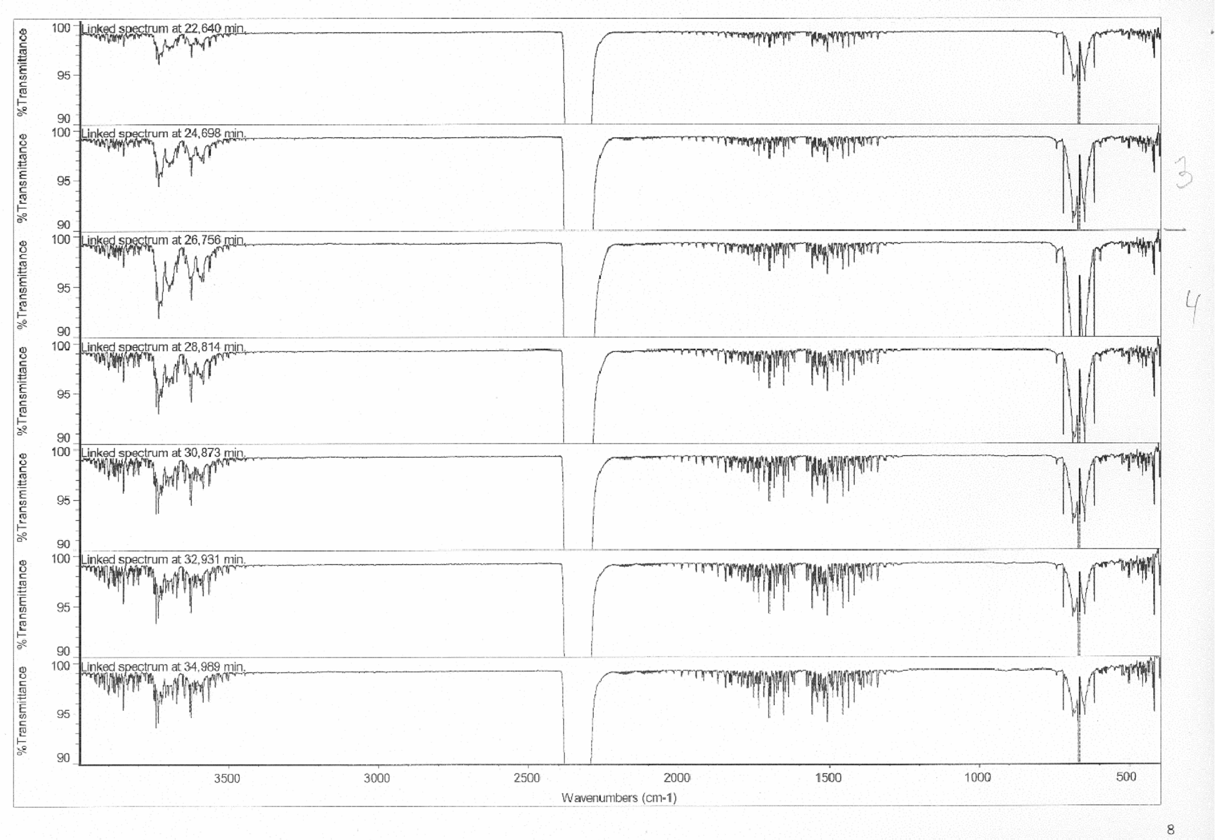

Linked spectrum at 22,640 min.
Linked spectrum at 24,698 min.
Linked spectrum at 26,756 min.
Linked spectrum at 28,814 min.
Linked spectrum at 30,873 min.
Linked spectrum at 32,931 min.
Linked spectrum at 34,989 min.
%Transmittance
100
95
90
3500
3000
2500
2000
1500
1000
500
Wavenumbers (cm-1)
3
4
8

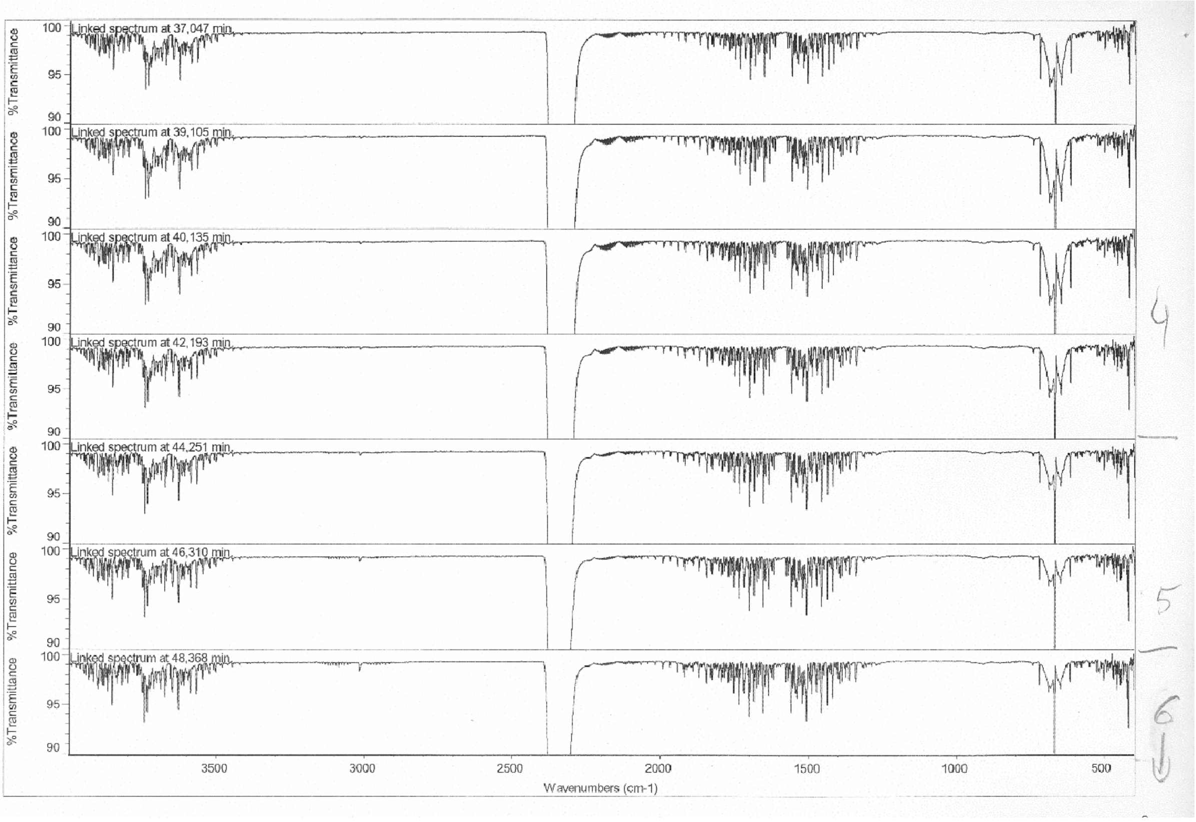

Linked spectrum at 37,047 min.
Linked spectrum at 39,105 min.
Linked spectrum at 40,135 min.
Linked spectrum at 42,193 min.
Linked spectrum at 44,251 min.
Linked spectrum at 46,310 min.
Linked spectrum at 48,368 min.
%Transmittance
Wavenumbers (cm-1)
4
5
6

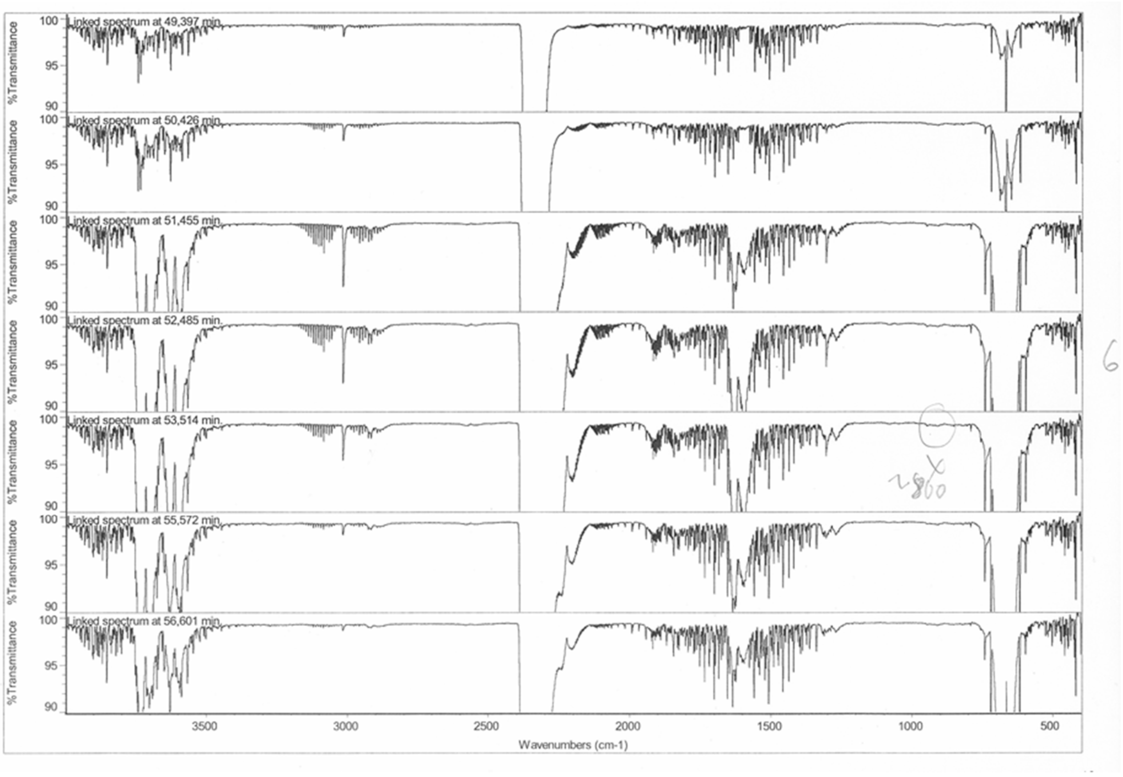
Linked spectrum at 49,397 min.
Linked spectrum at 50,426 min.
Linked spectrum at 51,455 min.
Linked spectrum at 52,485 min.
Linked spectrum at 53,514 min.
Linked spectrum at 55,572 min.
Linked spectrum at 56,601 min.
%Transmittance
100
95
90
3500
3000
2500
2000
1500
1000
500
Wavenumbers (cm-1)

Four selected FT-IR images to identify the evolved gases at Steps 4 (Setp IV, up) and (Step V, two at middle), and to the end of the experiment, at min. 95.7 (down).

See the non-identified gases (X) clearly detected at 850-950 cm^{-1} in the last spectrum, but also as very weak absorptions in the previous ones.

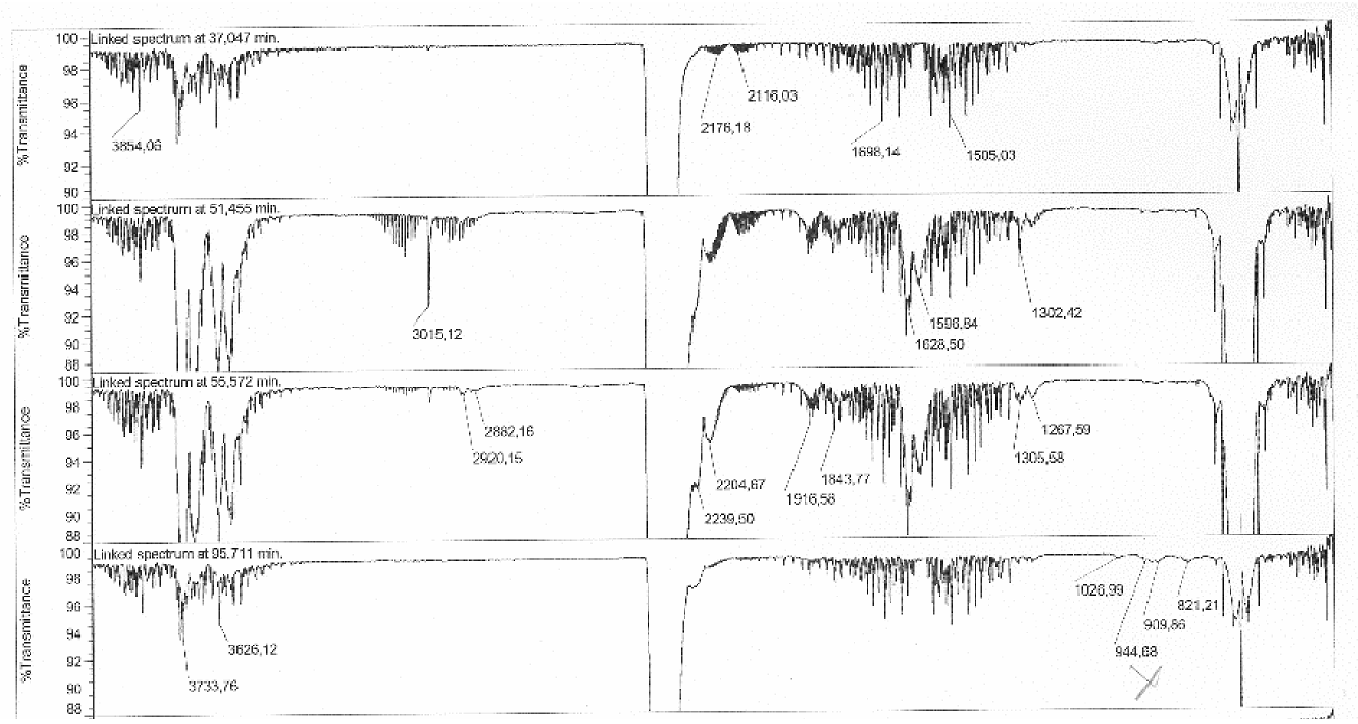

Plot of the FT-IR spectra, from the IR library, used for the identification of evolved gases during the TGA of compound 2.

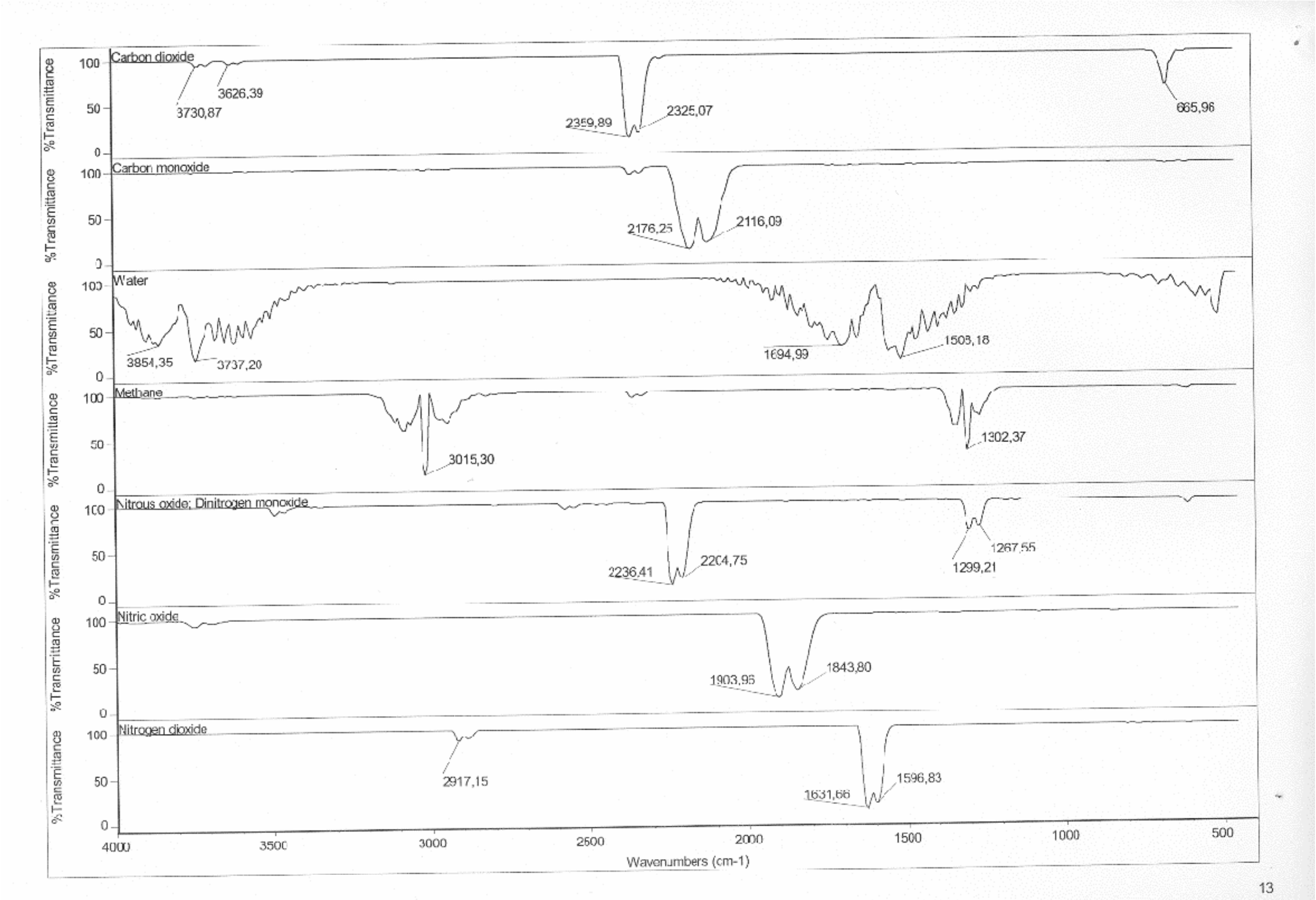

S.5 FT-IR spectra of solid samples for 1 and 2

S.5.1. FT-IR spectrum of compound 1

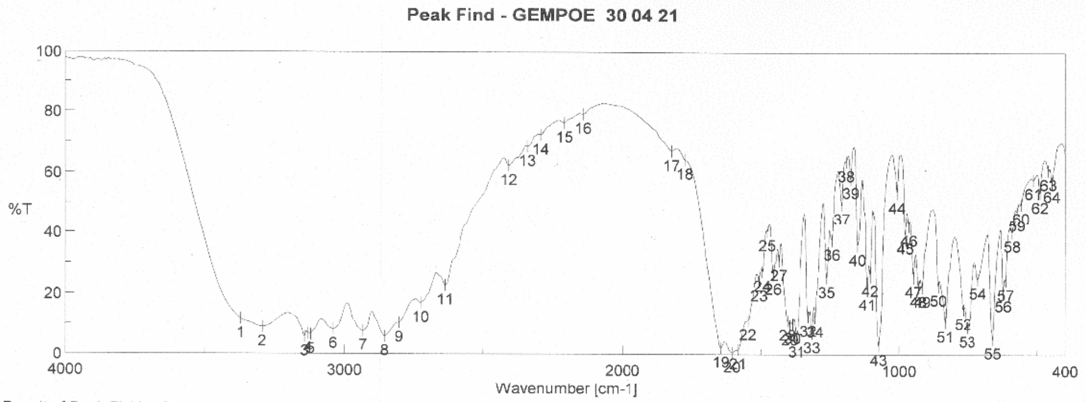

[Result of Peak Picking]

No.	Position	Intensity	No.	Position	Intensity	No.	Position	Intensity
1	3372.89	11.673	2	3295.75	8.98224	3	3142.44	5.71869
4	3128.94	6.87274	5	3120.26	6.49669	6	3040.23	8.24324
7	2934.16	7.702	8	2857.02	5.90015	9	2805.92	10.4292
10	2727.82	16.9691	11	2640.07	22.7651	12	2409.62	62.335
13	2343.09	68.5939	14	2295.84	72.307	15	2210.02	76.3109
16	2143.49	79.2407	17	1823.37	67.1157	18	1776.12	64.3888
19	1644.02	2.18843	20	1603.52	0.570778	21	1582.31	1.66484
22	1547.59	11.312	23	1508.06	24.1935	24	1496.49	27.3328
25	1479.13	40.5662	26	1456.96	26.3735	27	1436.71	30.9162
28	1405.85	11.1801	29	1397.17	9.61206	30	1387.53	10.1674
31	1372.1	5.63219	32	1328.71	12.4914	33	1315.21	7.0201
34	1303.64	12.239	35	1262.18	25.3111	36	1242.9	37.6026
37	1207.22	49.4807	38	1191.79	63.7116	39	1174.44	58.1804
40	1149.37	35.8319	41	1114.65	21.2614	42	1104.05	25.781
43	1073.19	2.89401	44	1007.62	53.2567	45	977.733	39.7218
46	965.198	42.2488	47	947.842	25.4917	48	930.485	22.2333
49	916.986	22.2272	50	858.168	22.6302	51	833.098	10.74
52	767.53	14.9127	53	753.066	9.13737	54	713.533	25.0153

S.5.2. FT-IR spectrum of compound 2

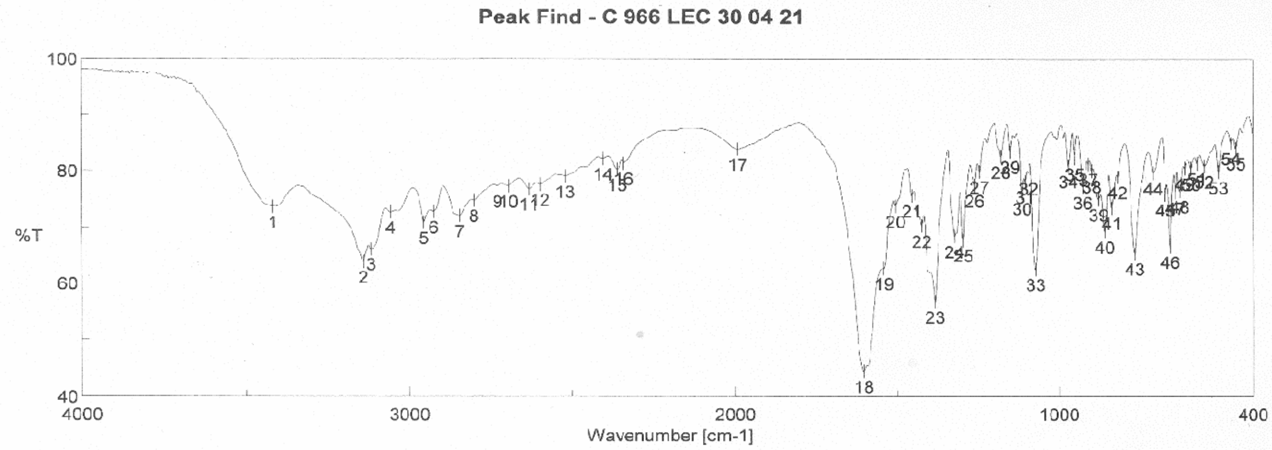

[Result of Peak Picking]

No.	Position	Intensity	No.	Position	Intensity	No.	Position	Intensity
1	3418.21	73.6543	2	3141.47	63.83	3	3118.33	66.12
4	3058.55	72.6712	5	2956.34	70.8563	6	2925.48	72.7412
7	2846.42	72.0682	8	2802.06	74.7368	9	2729.74	77.2519
10	2696.96	77.2708	11	2633.32	76.7439	12	2599.57	77.5467
13	2521.47	78.9798	14	2405.76	82.08	15	2361.41	80.1453
16	2342.12	81.2477	17	1992.11	83.7453	18	1604.48	44.4733
19	1542.77	62.6284	20	1506.13	73.6968	21	1455.99	75.5042
22	1425.14	70.2051	23	1384.64	56.679	24	1324.86	68.3724
25	1297.86	67.6667	26	1262.18	77.3562	27	1246.75	79.6971
28	1180.22	82.3197	29	1151.29	83.207	30	1114.65	75.9589
31	1105.01	77.9832	32	1094.4	79.4722	33	1073.19	62.4832
34	970.983	80.7116	35	951.698	82.0082	36	926.628	77.0021
37	909.272	81.0455	38	898.666	79.8277	39	877.452	74.8759
40	859.132	69.132	41	836.955	73.3848	42	818.634	78.7851
43	765.601	65.3002	44	707.747	79.4583	45	670.142	75.6986
46	655.679	66.5193	47	638.323	76.0541	48	624.823	76.3312
49	609.396	80.2488	50	590.111	80.2266	51	571.79	81.1566
52	548.649	80.6425	53	506.223	79.6343	54	467.653	84.7075

Complementary literature

1. Bellamy, L.J. (1975) The infrared spectra of complex molecules. 3rd Edition, Chapman and Hall, London. Chapters 13 (Amino-acids, their Hydrochlorides and Salts, and Amido-acids), 14 (Amines and Imines), 15 (Unsaturated Nitrogen Compounds).

2. Nakanishi, K. Infrared Absorption Spectroscopy – Practical. Holden-Day, Inc., San Francisco USA, and Nankodo Co. Ltd., Tokyo. 1962. Chapter 2. Tables of characteristic frequencies. The Table 7. Amines and Ammonium Salts). See, in particular, Table 7d (Amine Salts) in pag. 37.
Band(s) or group of relatively sharp band at 2700-2250 cm^{-1}.

3. Nakanoto, K. Infrared and Raman Spectra of Inorganic and Coordination Compounds. 6th Edition, Part B: Applications in Coordination, Organometallic and Bioinorganic Chemistry, Wiley & Sons, 2009. Part 1(Applications in Coordination Chemistry). 1.10. Complexes of Amino acids, EDTA and Related Ligands (pags. 67-74).
